# A dual specificity kinase, DYRK1A, as a potential therapeutic target for head and neck squamous cell carcinoma

**DOI:** 10.1038/srep36132

**Published:** 2016-10-31

**Authors:** Aneesha Radhakrishnan, Vishalakshi Nanjappa, Remya Raja, Gajanan Sathe, Vinuth N. Puttamallesh, Ankit P. Jain, Sneha M. Pinto, Sai A. Balaji, Sandip Chavan, Nandini A. Sahasrabuddhe, Premendu P. Mathur, Mahesh M. Kumar, T. S. Keshava Prasad, Vani Santosh, Geethanjali Sukumar, Joseph A. Califano, Annapoorni Rangarajan, David Sidransky, Akhilesh Pandey, Harsha Gowda, Aditi Chatterjee

**Affiliations:** 1 Institute of Bioinformatics, International Technology Park, Bangalore, 560 066, India; 2 Department of Biochemistry and Molecular Biology, Pondicherry University, Puducherry 605014, India; 3 Amrita School of Biotechnology, Amrita University, Kollam 690 525, India; 4 Manipal University, Madhav Nagar, Manipal 576104, India; 5 School of Biotechnology, KIIT University, Bhubaneswar 751024, India; 6 Department of Molecular Reproduction, Development and Genetics, Indian Institute of Science, Bangalore, 560012, India; 7 Department of Neuro-Virology, National Institute of Mental Health and Neurosciences, Bangalore 560029, India; 8 YU-IOB Center for Systems Biology and Molecular Medicine, Yenepoya University, Mangalore 575018, India; 9 Department of Pathology, National Institute of Mental Health and Neurosciences, Bangalore 560029, India; 10 Milton J. Dance Head and Neck Center, Greater Baltimore Medical Center, Baltimore, MD 21204, USA; 11 Department of Otolaryngology-Head and Neck Surgery, Johns Hopkins University School of Medicine, Baltimore, MD 21231, USA; 12 McKusick-Nathans Institute of Genetic Medicine,Johns Hopkins University School of Medicine, Baltimore, MD 21205, USA; 13 Department of Biological Chemistry, Johns Hopkins University School of Medicine, Baltimore, MD 21205, USA; 14 Department of Oncology, Johns Hopkins University School of Medicine, Baltimore, MD 21205, USA; 15 Department of Pathology, Johns Hopkins University School of Medicine, Baltimore, MD 21205, USA

## Abstract

Despite advances in clinical management, 5-year survival rate in patients with late-stage head and neck squamous cell carcinoma (HNSCC) has not improved significantly over the past decade. Targeted therapies have emerged as one of the most promising approaches to treat several malignancies. Though tyrosine phosphorylation accounts for a minority of total phosphorylation, it is critical for activation of signaling pathways and plays a significant role in driving cancers. To identify activated tyrosine kinase signaling pathways in HNSCC, we compared the phosphotyrosine profiles of a panel of HNSCC cell lines to a normal oral keratinocyte cell line. Dual-specificity tyrosine-(Y)-phosphorylation regulated kinase 1A (DYRK1A) was one of the kinases hyperphosphorylated at Tyr-321 in all HNSCC cell lines. Inhibition of DYRK1A resulted in an increased apoptosis and decrease in invasion and colony formation ability of HNSCC cell lines. Further, administration of the small molecular inhibitor against DYRK1A in mice bearing HNSCC xenograft tumors induced regression of tumor growth. Immunohistochemical labeling of DYRK1A in primary tumor tissues using tissue microarrays revealed strong to moderate staining of DYRK1A in 97.5% (39/40) of HNSCC tissues analyzed. Taken together our results suggest that DYRK1A could be a novel therapeutic target in HNSCC.

Squamous cell carcinoma of head and neck (SCCHN) is a common malignancy worldwide arising from various regions of upper-aero digestive tract and oral cavity. It is the sixth most common cancer worldwide^1^. Approximately more than 500,000 new cases and 12,000 deaths are estimated annually in United States for head and neck cancer^2^. The major risk factors in HNSCC include smoking, alcohol consumption and human papillomavirus (HPV) infections. Despite all the treatment strategies, therapeutic resistance/failure and tumor recurrence still exists making the five-year survival rate, sub-optimal^3^. Hence it is important to understand the molecular events associated with HNSCC for the identification of novel therapeutic targets.

Protein kinases are the key regulators of signal transduction pathways in many cellular processes. Aberrant activation of kinase driven pathways has been reported to play a crucial role in multiple cellular processes that leads to cancer progression. Such alterations can be assessed by studying the proteome through analysis of the phosphoproteome. In recent years, kinases have become one of the most intensively studied groups of proteins as drug targets. To date, 28 small molecule kinase inhibitors have been approved by FDA for cancer therapy^4^. Identification of imatinib, a small molecule inhibitor against BCR-ABL tyrosine kinase, by Druker and colleagues revolutionized the treatment of patients with chronic myeloid leukemia^5^,^6^. Although targeted therapy using EGFR specific antibody cetuximab, is used in the treatment of HNSCC; non-responsiveness and development of resistance is a common hindrance^7^. Protein kinases not only play a central role in cell signaling networks but also serve as excellent therapeutic targets. Phosphoproteome profiling to identify activated kinase pathways is an established approach to identify novel therapeutic targets in cancer^8^. To achieve this, we studied the activation of signaling molecules in a panel of HNSCC cell lines and a normal oral keratinocyte cell line (OKF6/TERT1) using phosphoproteomics approach. We identified a total of 38 proteins which included multiple kinases which were found to be differentially phosphorylated in all the HNSCC cell lines compared to the normal oral keratinocyte cell line, OKF6/TERT1. Dual-specificity tyrosine-(Y)-phosphorylation regulated kinase 1A (DYRK1A) was one of the identified kinases which showed hyperphosphorylation (fold change ≥1.5) in all the 6 HNSCC cell lines compared to normal oral keratinocytes.

DYRK1A belongs to dual specificity tyrosine (Y) phosphorylation regulated kinase (DYRK) family which is known to be activated through autophosphorylation of tyrosine residues in the activation loop and phosphorylates their substrates on serine and threonine residues^9^. Other members of this family include DYRK1B, DYRK2, DYRK3, DYRK4A and DYRK4B. Studies have revealed that DYRK family kinases play an important role in regulating cell proliferation and apoptosis^10^,^11^. DYRK1A has been reported to be strongly expressed in the brain and known to regulate various functions in brain^12^. However, studies by other groups have reported overexpression of DYRK1A, and its closest member DYRK1B, in various tumors including glioblastoma, ovarian cancer, lung cancer, colon cancer and pancreatic cancer^13^,^14^,^15^,^16^,^17^ suggesting a role of this molecule in tumorigenesis. A study by Pozo *et al*., showed that inhibition of DYRK1A stimulated EGFR degradation and reduced EGFR-dependent tumor growth in glioblastoma^13^. DYRK1A plays an important role in cell survival by phosphorylating caspase 9 at Thr-125 and inhibiting its action in apoptosis^11^. Taken together these studies indicate that DYRK1A plays a significant role in mediating survival of cancer cells. Although the role of DYRK1A in cancers has been characterized, its role in HNSCC is not defined. In this study, we have assessed the role of DYRK1A as a potential therapeutic target in HNSCC.

## Results

### Quantitative phosphotyrosine analysis of HNSCC

**Figure 1:**
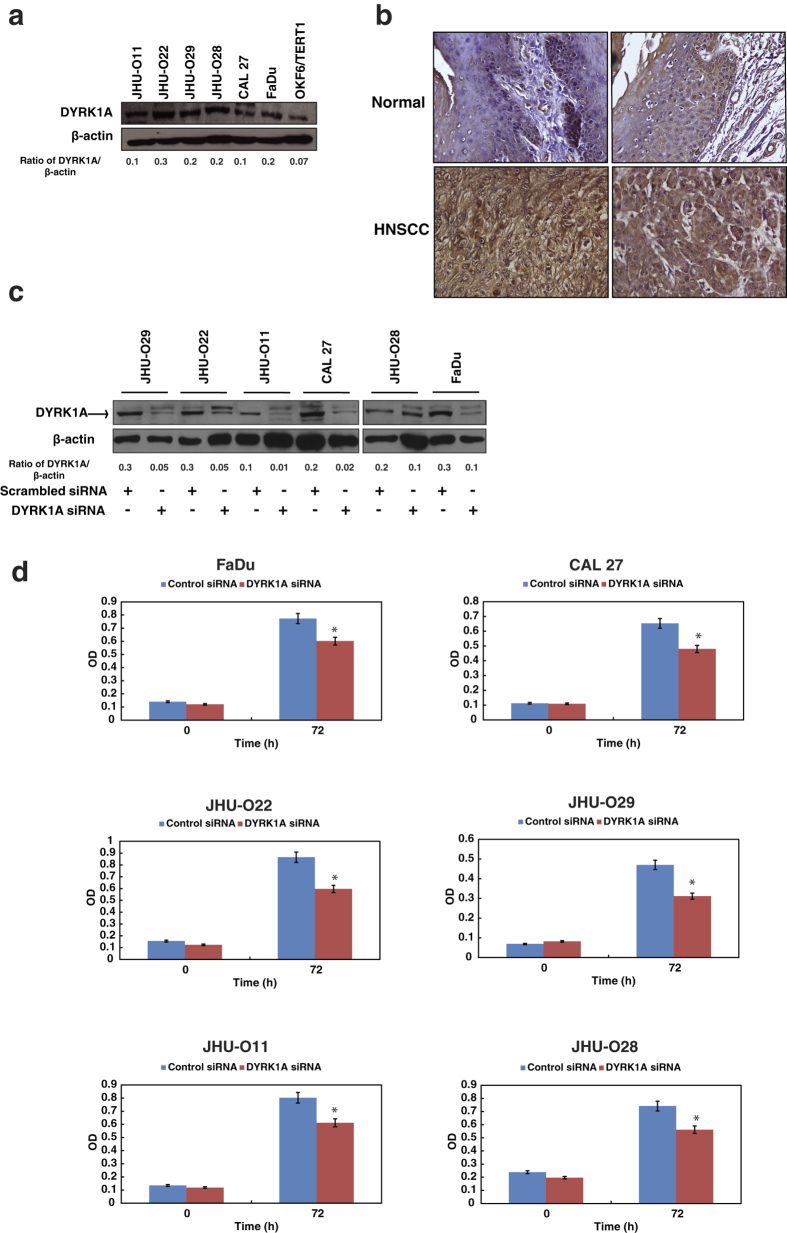
Inhibition of DYRK1A reduces cellular proliferation in HNSCC. (**a**) Western blot analysis shows the expression profile of DYRK1A in a panel of HNSCC cell lines – JHU-O11, JHU-O22, JHU-O28, JHU-O29, FaDu and CAL 27 compared to normal oral keratinocytes OKF6/TERT1. (**b**) Immunohistochemical validation of DYRK1A in HNSCC tissue - representative sections from normal and HNSCC cases were stained with anti-DYRK1A antibody. (**c**) Western blot analysis depicting DYRK1A expression in HNSCC cell lines upon transfection with DYRK1A siRNA. β-actin was used as a loading control. (**d**) Cellular proliferation of HNSCC cells upon siRNA mediated silencing of DYRK1A (*p < 0.05).

We employed tandem mass tag (TMT)-based labeling technology coupled with anti-phosphotyrosine antibody-based enrichment approach to identify differentially phosphorylated proteins between normal oral keratinocyte OKF6/TERT1, and a panel of HNSCC cell lines (JHU-O11, JHU-O22, JHU-O28, JHU-O29, FaDu and CAL 27) (Supplementary Fig. 1). We identified a total of 51 phosphosites in 38 proteins in the HNSCC cells compared to OKF6/TERT1. Amongst the hyperphosphorylated proteins, we identified molecules including protein tyrosine phosphatase, non-receptor type 11 (PTPN11), myelin protein zero-like 1 (MPZL1) and tyrosine kinases such as LYN proto-oncogene (LYN), EPH receptor A2 (EPHA2) and DYRK1A. The overexpression of PTPN11, LYN and EPHA2 has been reported in head and neck cancer^18^,^19^,^20^. Tyr-321 is the known activation site of DYRK1A^21^. We identified hyperphosphorylation of DYRK1A at Tyr-321 in all HNSCC cell lines. As expected, protein phosphorylation pattern was heterogeneous across HNSCC cell lines (Table 1). Western blot analysis revealed overexpression of DYRK1A in HNSCC cell lines compared to OKF6/TERT1 (Fig. 1a).

### Immunohistochemical validation of DYRK1A in HNSCC tissue

Our western blot results revealed overexpression of DYRK1A in HNSCC cell lines. We checked the expression of DYRK1A in primary HNSCC tissues. Tissue microarray-based immunohistochemical validation was carried out using 40 HNSCC tissues. A variable staining pattern was noted across cases of HNSCC. 97.5% (39 of 40) of HNSCC cases showed moderate to strong staining (1+ to 2+) while 2.5% (1 of 40) of the cases showed negative staining. A Chi-square test clearly indicated a significant overexpression of DYRK1A in HNSCC cases (p-value = 0.0076). The results of the immunohistochemical validation are provided in Table 2. The representative staining patterns for DYRK1A in HNSCC and adjacent normal tissues are illustrated in Fig. 1b.

### Inhibition of DYRK1A reduces cellular proliferation in HNSCC

Since DYRK1A was found to be overexpressed in all the HNSCC cell lines, we next studied the role of DYRK1A in cell proliferation. Cellular proliferation for the panel of HNSCC cell lines was studied after silencing of endogenous expression of DYRK1A using its specific siRNA. Western blot analysis confirmed efficient knockdown of DYRK1A in all HNSCC cells (Fig. 1c). We further assessed the effect of DYRK1A silencing on cell proliferation of HNSCC cell lines. A decrease in cellular proliferation of HNSCC cells was observed upon silencing of DYRK1A (Fig. 1d). Akin to siRNA results, inhibition of DYRK1A using its specific inhibitor harmine^22^ also led to decrease in the cellular proliferation of majority of the HNSCC cells (Supplementary Fig. 2).

### Inhibition of DYRK1A reduces the colony forming ability of HNSCC cells

**Figure 2:**
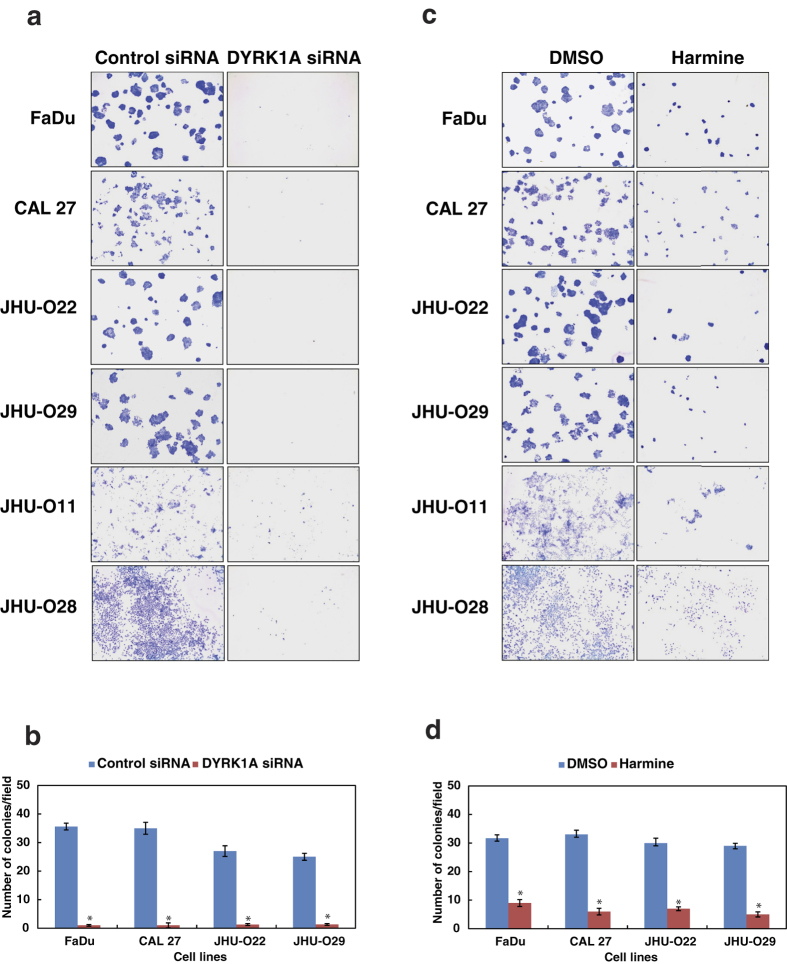
Inhibition of DYRK1A affects the colony forming ability of the HNSCC cells. (**a**) Colony formation assay following siRNA mediated knockdown of DYRK1A or control siRNA in a panel of HNSCC cell lines, as indicated. Colonies formed were visualized after staining with methylene blue. (**b**) A graphical representation of the colony forming ability of the HNSCC cells upon DYRK1A silencing (*p < 0.05). (**c**) Colony forming ability of the HNSCC cells upon inhibition of DYRK1A using harmine or control (DMSO), in the indicated panel of HNSCC cells. (**d**) A graphical representation of the colony forming ability of HNSCC cells upon harmine treatment (*p < 0.05). Representative images were photographed at a magnification (2.5x).

Having observed that DYRK1A plays an essential role in cellular proliferation, we next studied the role of DYRK1A in the colony forming ability of the HNSCC cells. siRNA mediated silencing of DYRK1A resulted in a decrease in the colony forming ability of the HNSCC cells (Fig. 2a,b). In concordance with the siRNA results, inhibition of DYRK1A using harmine in HNSCC cell lines resulted in a significant decrease in the colony formation ability of the cells (Fig. 2c,d).

### Inhibition of DYRK1A reduces the invasive ability of the HNSCC cells

**Figure 3:**
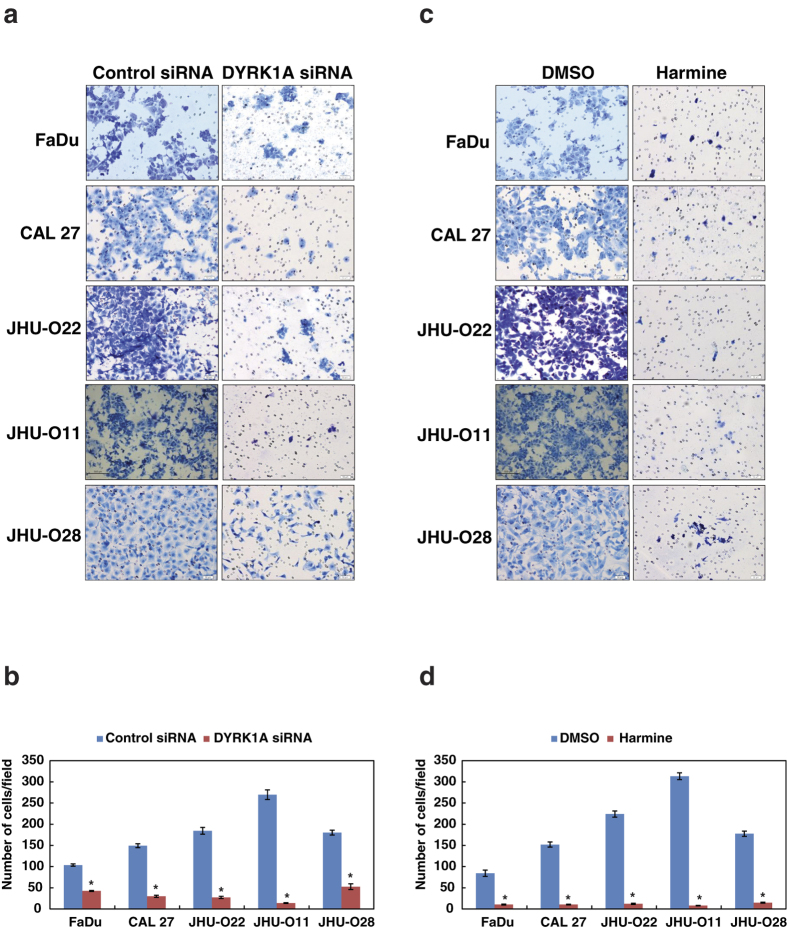
Inhibition of DYRK1A reduces the invasive ability of the HNSCC cells. (**a**) HNSCC cells were transfected with DYRK1A specific siRNA and/or scramble siRNA and invasion assays were carried out using in a transwell system using Matrigel-coated filters and the number of cells that migrated to the lower chamber was counted. Cells that migrated are visualized following methylene blue staining in a panel of HNSCC cell lines as indicated and invaded cells were photographed. (**b**) Graphical representation of invasive ability of HNSCC cells upon DYRK1A silencing (*p < 0.05). (**c**) HNSCC cells were treated with DYRK1A inhibitor (harmine) or vehicle control (DMSO) and invaded cells were photographed. (**d**) Graphical representation of invasive ability of DYRK1A upon inhibition with harmine (*p < 0.05). Representative images were photographed at a magnification (10x).

Since inhibition of DYRK1A led to a decrease in the colony formation ability of the HNSCC cell lines, we next studied if DYRK1A has a potential role in HNSCC invasiveness. We investigated the *in vitro* invasive capabilities of the HNSCC cells using Matrigel invasion assay. siRNA mediated silencing of DYRK1A, showed decrease in invasive property of all the HNSCC cells (Fig. 3a,b). In agreement with the siRNA results, inhibition of DYRK1A with harmine, resulted in a significant decrease in the invasive property of all the HNSCC cells (Fig. 3c,d). Taken together, our results indicate that DYRK1A may play an essential role in HNSCC metastasis.

### Inhibition of DYRK1A suppresses tumor growth *in vivo*

**Figure 4:**
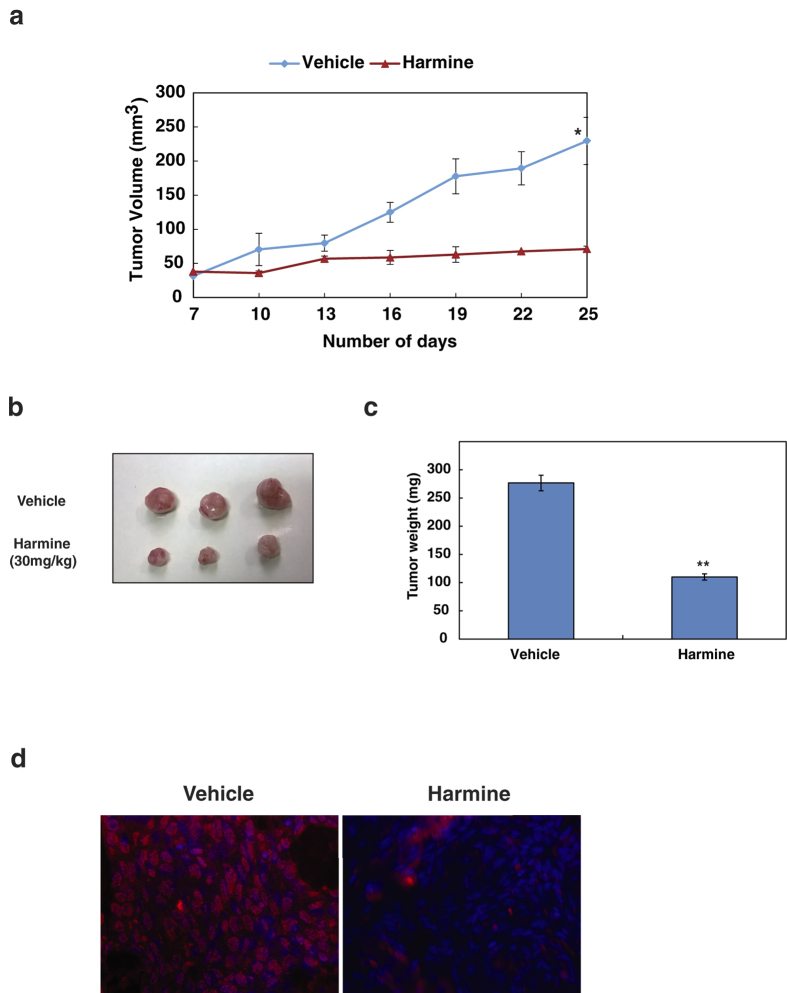
Inhibition of DYRK1A suppresses tumor growth *in vivo* (**a**) CAL 27 (2 × 10^6^) cells were injected into the flanks of female nude mice (n = 10) and tumor growth kinetics is represented for a period of 25 days. *p = 0.02 (**b**) Representative pictures of tumors from vehicle and harmine treated groups. (**c**) Bar graph representing the tumor weights (**p < 0.05). (**d**) Expression of Ki67 (Alexa Fluor 594) in xenograft tissue sections was determined by immunofluorescence. Cell nuclei were stained blue with 4′,6-diamidino-2-phenylindole (DAPI).

Having observed DYRK1A affects both HNSCC cellular proliferation and invasive potential *in vitro*, we next studied the oncogenic potential of DYRK1A by targeting DYRK1A *in vivo*. Athymic nude mice were injected subcutaneously (s.c.) with CAL 27 cells. At day 7, when the tumors reached the size of approximately 50 mm^3^, mice were randomized into two groups of five animals each and treated with either vehicle alone (DMSO) or harmine (15 mg/kg/injection, every 3 days till 3 weeks) intraperitoneally (i.p.). Tumor size was measured every 3 days and the mean tumor volume was calculated. We observed significant differences in tumor growth between vehicle control and harmine treated group over a 25-day experimental period (Fig. 4a). The mice were sacrificed at the end of 25 days and tumors extracted from harmine treated group had significant lower tumor mass compared to vehicle group (Fig. 4b,c). Further we examined the expression of proliferation marker, Ki67 in xenograft sections using immunofluorescence. The data revealed a decrease in expression of Ki67 in harmine treated xenograft tissue compared to vehicle treated tissue (Fig. 4d).

### Inhibition of DYRK1A induces apoptosis *in vitro* and *in vivo*

**Figure 5:**
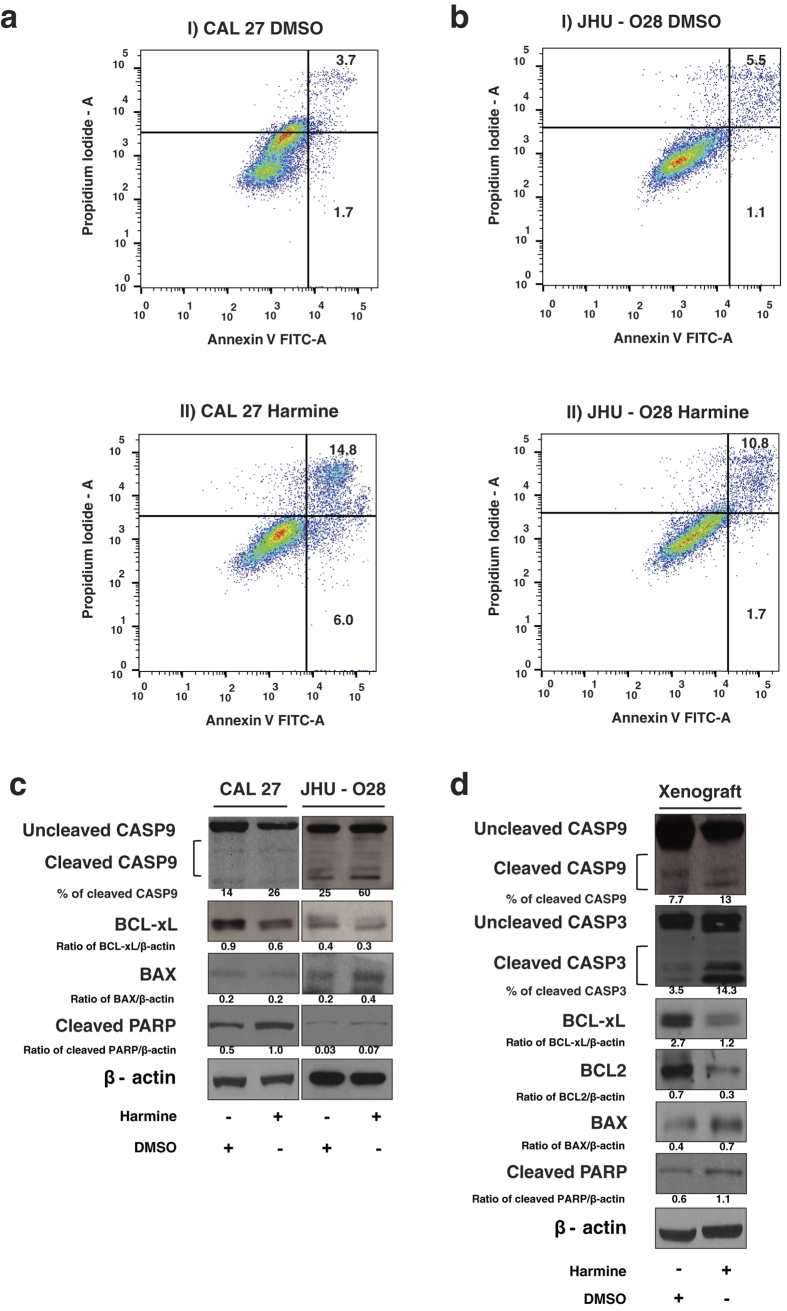
Inhibition of DYRK1A induces apoptosis *in vitro* and *in vivo*. Apoptosis was measured in CAL 27 (**a**) and JHU-O28 (**b**) cells using Annexin V/PI staining. (**c**) Western blot analysis was carried out for the indicated proteins using CAL 27 and JHU-O28 cells treated with harmine or DMSO (control). β-actin was used as a loading control. (**d**) Western blot analysis was carried out using cellular lysates of xenograft tissue (Harmine and DMSO treated) for the indicated proteins. β-actin was used as a loading control.

Next we studied the role of inhibition DYRK1A in apoptosis in HNSCC cells. CAL 27 and JHU – O28 were treated with harmine or DMSO (control) and apoptosis was determined by staining cells using annexin V fluorescein isothiocyanate and propidium iodide (PI). Harmine treatment induced 14.8% late apoptosis (both annexin V and PI positive) in CAL 27 cells, with 6.0% of cells undergoing early apoptosis (annexin V positive and PI negative), compared to 3.7% late apoptotic cells and 1.7% of early apoptotic cells in the control (DMSO treated) cells (Fig. 5a). In JHU-O28 cells harmine treatment induced 10.8% and 1.7% of late and early apoptosis respectively compared to 5.5% and 1.1% late and early apoptotic cells respectively in the control (DMSO) treated cells (Fig. 5b). In addition, we examined the expression of pro and anti-apoptotic proteins upon inhibition of DYRK1A in CAL 27 and JHU-O28 cells. Western blot analyses revealed a decrease in BCL-xL and an increased expression of BAX upon inhibition of DYRK1A with harmine (Fig. 5c). Treatment with harmine also resulted in the activation of CASP9 (Caspase-9) and PARP (Poly (ADP-ribose) polymerase) in both CAL 27 and JHU-O28 cells (Fig. 5c). Further we studied the expression of pro and anti-apoptotic proteins in the xenograft tissue treated with either vehicle control (DMSO) or harmine. Western blot analysis revealed a decrease in the expression of both BCL-xL and BCL2 and an increased expression of pro-apoptotic protein BAX (Fig. 5d). *In vivo* treatment with harmine also promoted the activation of CASP9, CASP3 (Caspase 3) and PARP indicating induction of apoptosis (Fig. 5d). These results indicate that inhibition of DYRK1A leads to induction of apoptosis in HNSCC cells.

### Inhibition of DYRK1A leads to activation of FOXO3A in HNSCC

**Figure 6:**
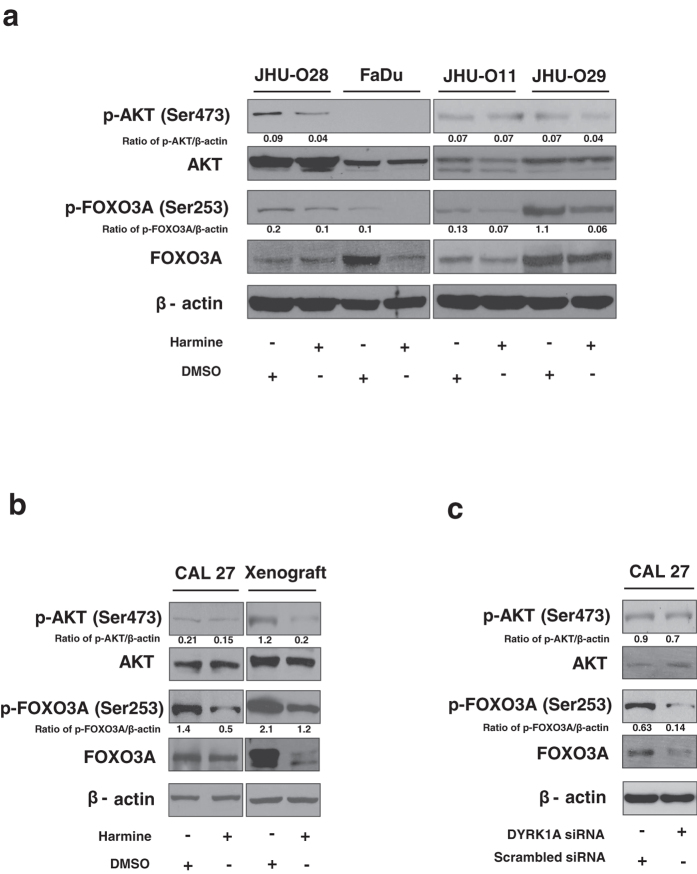
Inhibition of DYRK1A leads to activation of FOXO3A in HNSCC. HNSCC cell lines JHU-O28, FaDu, JHU-O11, JHU-O29 (**a**) and CAL 27 (**b**) were treated with DYRK1A inhibitor harmine. Immunoblot analysis of p-AKT (Ser473), Total AKT, p-FOXO3A (Ser253) and Total FOXO3A was performed. β-actin was used as loading control. (**b**) Western blot analysis was performed using cellular lysates from xenograft tissue (Harmine and DMSO treated) for AKT (Ser473), Total AKT, p-FOXO3A (Ser253) and Total FOXO3A. (**c**) CAL 27 cells were treated with DYRK1A siRNA or control siRNA and Western blot analysis was carried for the indicated proteins.

Since our *in vitro* and *in vivo* results showed that DYRK1A plays an essential role in the oncogenic potential of HNSCC, we sought to study the signaling mechanisms of DYRK1A in HNSCC. Previous studies have shown activation of AKT and MAPKs in the brain of DYRK1A-overexpressing mice^23^. It has also been reported that DYRK1A phosphorylates forkhead transcription factors and mediates cell survival^24^,^25^. FOXO3A is a known substrate of AKT and MAPK and studies have shown that phosphorylation of FOXO3A by AKT and MAPKs are inhibitory and helps in the survival of cancer cells^26^,^27^. Inhibition of DYRK1A using harmine decreased p-AKT levels without affecting the total AKT levels in three of the HNSCC cell lines studied (Fig. 6a,b). As AKT is reported to phosphorylate FOXO3A on Ser253^28^, we examined the effect of DYRK1A-AKT pathway on FOXO3A phosphorylation. Inhibition of DYRK1A decreased phosphorylation of FOXO3A on Ser253 in all HNSCC cell lines (Fig. 6a,b). Western blot analysis of the xenograft tissue (treated with harmine) also showed a decreased expression of p-AKT and p-FOXO3A (Fig. 6b). We further analysed the levels of p-AKT (Ser473) and p-FOXO3A (Ser253) in CAL 27 cell line treated with DYRK1A siRNA. We observed a decreased phosphorylation of both p-AKT (Ser473) and p-FOXO3A (Ser253) in the siRNA treated cells (Fig. 6c). These results indicate that DYRK1A inhibition leads to activation of FOXO3A (decrease in phosphorylation) in HNSCC cells.

## Discussion

We and others have demonstrated that phosphoproteomics is an effective technique to study aberrantly activated kinase signaling pathways in multiple malignancies^8^,^29^,^30^. There is substantial literature indicating that protein kinases are most frequently involved in tumorigenesis^31^. It is now well established that most cancers are heterogeneous and that specific kinases are hyperactive in at least a subset of a given cancers and drive their progression. Studies by us and others suggest that these activated kinases can serve as both surrogate markers for monitoring responses and also serve as potential therapeutic targets^32^,^33^.

To identify novel therapeutic targets in HNSCC, we studied the phosphotyrosine signaling in a panel of HNSCC cell lines and normal oral keratinocyte cell line OKF6/TERT1. This led to the identification of hyperphosphorylated kinases such as YES proto-oncogene 1, Src family tyrosine kinase (YES1), epidermal growth factor receptor (EGFR), LYN proto-oncogene (LYN), EPH receptor A2 (EPHA2) and DYRK1A in the HNSCC cell lines. DYRK1A is a member of the conserved family of DYRKs that autophosphorylate tyrosine in their activation loop^34^. Depending upon the cellular context, DYRK1A is known to function both as a tumor suppressor and an oncogene^35^. Recent studies have indicated the role of DYRKs in regulation of mitotic transition and apoptosis induced by DNA damage^36^. Various members of DYRK family have been reported to play key role in cell proliferation and survival in cancer cell lines. Pharmacological inhibition of DYRK1A in mouse xenograft based studies has demonstrated it as a potential target in Glioblastoma^13^. DYRK1A has been shown to play role in quiescence by the assembly of DREAM complex and inhibits cell proliferation^37^. Our data illustrates the role of DYRK1A in both proliferation and metastatic potential in HNSCC. Silencing and/or inhibition of DYRK1A using siRNA or inhibitor (harmine) showed an increased apoptosis with significant reduction in cellular proliferation, invasion and colony forming ability of the HNSCC cells. We observed a significant decrease in tumor load in mouse xenografts upon DYRK1A inhibition. Our immunohistochemical studies indicate strong to moderate expression of DYRK1A in 97.5% of HNSCC primary tissue. These results suggest that DYRK1A has the potential to become a novel therapeutic target for HNSCC.

It has been reported that DYRK1A activates PI3K/AKT and MAPK pathways in conditions such as hyperhomocystenimia^23^, however the connecting link between these major signaling pathways and DYRK1A remains unclear in cancer. It has been reported that FOXO transcription factors can be targeted by DYRK kinases^24^. FOXO transcription factors are known to play a role in regulating genes responsible for apoptosis and cell cycle progression^38^. They are mainly regulated by phosphorylation, acetylation and ubiquitination. Previous studies have shown that phosphorylation of FOXO3A by AKT and MAPK1/2 promotes cellular survival^26^,^27^. We studied the effect of DYRK1A inhibition in HNSCC cell lines. Our study demonstrates that inhibition of DYRK1A led to cell death via decrease in phosphorylation of FOXO3A which is both dependent and independent on AKT signaling in HNSCC cells. Taken together our data suggest the role of DYRK1A signaling in promoting cellular survival in HNSCC.

In summary this study shows that DYRK1A was hyperphosphorylated and overexpressed in HNSCC cells. We showed a decrease in oncogenic characteristics including proliferation, invasion and migration of cells by inhibition of DYRK1A using *in vitro* assays. Inhibition of DYRK1A *in vivo* also resulted in a reduction in tumor growth. Our results indicate that DYRK1A plays a crucial role in regulating major signaling pathways in HNSCC. These results suggest that DYRK1A plays an important role in carcinogenesis and can serve as a potential therapeutic target in HNSCC. Future investigations in larger patient cohorts are required to confirm our findings in clinical settings. Our work provides a scaffold for future studies to systematically investigate the role of DYRK1A in HNSCC.

## Methods

### Cell culture

FaDu and CAL 27 were purchased from ATCC. Normal oral keratinocyte OKF6/TERT1 was a gift from Dr. James Rheinwald at Brigham and Women’s Hospital in Boston, MA. JHU-O11, JHU-O22, JHU-O28, JHU-O29 and FaDu were cultured in RPM1-1640 media supplemented with 10% fetal bovine serum and 1% penicillin/streptomycin. CAL 27 cells were cultured in DMEM medium supplemented with 10% fetal bovine serum and 1% penicillin/streptomycin. OKF6/TERT1 was cultured and maintained in KSFM (keratinocyte-serum free media) (Life Technologies, Grand Island, NY) supplemented with bovine pituitary extract (25 mg/ml), calcium chloride (0.4 mM), epidermal growth factor (0.2 ng/ml) and 1% penicillin/streptomycin. All cell lines were grown in a humidified incubator with 5% CO_2_ at 37 °C. The cell lines used for the study were authenticated by short tandem repeat analysis at the Genetic Resources Core Facility of Johns Hopkins University School of Medicine.

### Sample Preparation for LC-MS/MS analysis

#### Protein isolation and digestion

Each cell line was grown to 70% confluence and then maintained in serum free medium for 12 h before the cells were harvested for protein isolation. The cells were lysed in lysis buffer (2% SDS, 5mM sodium fluoride, 1 mM β-glycerophosphate, 1mM sodium orthovanadate in 50mM Triethyl ammonium bicarbonate (TEABC)). Protein concentration was estimated using BCA (Pierce, Waltham, MA) assays. Equal amount of protein from each cell line was used for protein digestion. We employed filter aided sample preparation (FASP) protocol using 30kDa filters to reduce the amount of SDS in the lysate^39^. The protein sample was reduced and alkylated as described previously^40^. Trypsin digestion was carried using TPCK treated trypsin (1: 20) for 12–16 h at 37 °C.

#### TMT labeling and phosphopeptide enrichment

Peptide samples were labeled using TMT 10 plex reagents (Thermo Scientific, Bremen, Germany) as described previously^41^. Peptides derived from all OKF6/TERT1 were labeled with TMT tag 127N, JHU-O28 with 128C, JHU-O11 with 129N, FaDu with 129C, CAL 27 with 130N, JHU-O22 with 130C and JHU-O29 with 131. The labeled samples were pooled and loaded on to a Sep-Pak C_18_ (Waters) column equilibrated with 0.1% TFA. The column was further washed with 0.1% TFA and the peptides were eluted in 6 ml of 40% ACN with 0.1% TFA. Peptides were lyophilized and subjected to phosphopeptide enrichment. The enrichment of tyrosine phosphorylated peptides from TMT labeled lysates was carried out using Phosphoscan Kit (P-Tyr-1000, Cell signaling technology, Danvers, MA) as described previously^29^.

### LC-MS/MS analysis

LTQ-Orbitrap Velos mass spectrometer interfaced with Proxeon Easy nLC system (Thermo Scientific, Bremen, Germany) was used for the analysis of TMT-labeled samples. The MS parameters used for analysis were followed as described previously^40^. Briefly, precursor MS scan (from m/z 350–1,700) and MS/MS was acquired with a mass resolution of 60,000 and 30,000 at 400 m/z in orbitrap mass analyzer. In each duty cycle ten most intense peaks were selected for MS/MS fragmentation using higher-energy collision dissociation mode at 45% normalized collision energy and isolation width was set to 1.9 m/z.

### Data analysis

The Proteome Discoverer (Version 1.4.1.14) software suite (Thermo Scientific, Bremen, Germany) was used to carry out protein identification and quantitation. Mass spectrometry data was searched against NCBI Human RefSeq protein database (Version 65) supplemented with common contaminants using Mascot (version 2.2.0) and SEQUEST search algorithms. The search parameters included trypsin as the protease with maximum of 2 missed cleavage allowed; oxidation of methionine and phosphorylation of tyrosine, serine and threonine was set as dynamic modifications while static modifications included carbamidomethylation at cysteine and TMT modification at N-terminus of the peptide and lysine. Precursor mass tolerance was set to 10 ppm and fragment mass tolerance was set to 0.05 Da. The false discovery rate (FDR) was calculated by carrying out decoy database searches and peptides scoring better than 1% FDR score cut-off were considered for further analysis^42^. The ratios were calculated by the quantitation node and the phosphorylation probability was calculated using phosphoRS node in Proteome Discoverer.

### Data availability

The mass spectrometry data is publicly available and accessible to other researchers. The complete mass spectrometry data generated from this study has been deposited to the ProteomeXchange (http://www.proteomexchange.org) via PRIDE partner repository with the dataset identifier PXD002132. The immunohistochemistry data was submitted to Human Proteinpedia^43^ and can be visualized with the link http://www.humanproteinpedia.org/Experimental_details?exp_id=TE-552658 for adjacent non-neoplastic tissue and http://www.humanproteinpedia.org/Experimental_details?can_id=105424 for tumor tissues.

### siRNA Transfection

ON-TARGETplus SMARTpool control siRNA and DYRK1A siRNA were purchased from Dharmacon (Lafayette, CO) and cells were transfected with control and DYRK1A siRNA using RNAiMAX reagent (Invitrogen, Carlsbad, CA) according to the manufacturer’s instructions.

### Cell proliferation assays

Cell proliferation assays was carried out as described previously with minor modifications^40^. The HNSCC cells were seeded at a density of 8000 cells/well into 96-well plate and treated with DYRK1A siRNA or its inhibitor harmine (20 μM) for 72 h. Cell proliferation was determined using MTT (3-(4, 5-dimethylthiazol-2yl)-2, 5-diphenyl tetrazolium bromide) assays as described^44^. Absorbance was measured at 570 nm and 650 nm. All experiments were carried out in triplicates.

### Colony formation assays

Colony formation assays were carried out as described previously with minor modifications^45^. HNSCC cell lines were seeded at a cell density of 8000 cells per well of a 6-well plate and transfected with either DYRK1A siRNA or control siRNA. After 48 h of transfection, cells were seeded into 6 well plates. Cell colonies were allowed to grow for 10–14 days. The colonies formed were fixed with methanol and stained with 4% methylene blue. Similarly, colony formation was done in the presence of harmine, DYRK1A inhibitor. Colonies formed was counted for ten randomly selected viewing fields and representative images were photographed at 2.5x magnification. All experiments were carried out in triplicate.

### Cell invasion assays

Invasion assays were performed in a transwell system (BD Biosciences) with Matrigel-coated filters, and cellular invasion was evaluated after 48 h as described previously^45^. Briefly, invasiveness of the cells was assayed in the membrane invasion culture system using polyethylene terephthalate (PET) membrane (8-μm pore size) in the upper compartment of a transwell coated with Matrigel (BD BioCoat Matrigel Invasion Chamber; BD Biosciences). The cells were transfected with either control or DYRK1A siRNA and seeded at 2.0 × 10^4^ cells per 500 μl of media on the Matrigel-coated PET membrane in the upper compartment. The lower compartment was filled with complete growth media and the plates were maintained at 37 °C for 48 h. At the end of the incubation time, the upper surface of the membrane was wiped with a cotton-tip applicator to remove non migratory cells. Cells that migrated to bottom side of membrane were fixed and stained using 4% methylene blue. The number of cells that penetrated was counted for ten randomly selected viewing fields at 10x magnification. Similarly, invasion assay was carried out in the presence of harmine. Each measurement was performed in duplicate and experiments were repeated three times.

### *In vivo* studies

Subcutaneous xenografts were generated in 6-week-old athymic ^nu/nu^ female nude mice with CAL 27 (2 × 10^6^) cells in both the flanks of mice. One week following tumor cell inoculation, ten mice with successfully engrafted CAL 27 xenografts (50 mm^3^) were randomized into two cohorts of five animals per group and were treated with either DMSO (Vehicle) or harmine (15 mg/kg/injection, every 3 days) intraperitoneally (i.p.). Harmine dosage was selected based on previous study^13^. Tumor size was measured after every 3 days using digital calipers (Fisher Scientific) upto four weeks and the mean tumor volume was calculated using the formula (π/6(d1xd2)^3/2^)^46^. All the animal experiments were approved by the Institutional Animal Care and Use Committee of Indian Institute of Sciences, Bangalore, India. All the experiments were performed in accordance with the relevant guidelines and regulations.

### Immunohistochemical assays

Immunohistochemical analysis using commercially available tissue microarray was carried out for DYRK1A. TMAs obtained commercially were processed and immunohistochemical staining was carried out as described previously^47^. Briefly, the formalin fixed paraffin embedded tissue sections were deparaffinised prior to antigen retrieval by addition of antigen retrieval buffer (citrate buffer) for 20 min. Action of endogenous peroxidases was quenched using blocking solution (methanol and H_2_O_2_ mixed at 3:1 ratio) and then washed with wash buffer (phosphate buffered saline). Polyclonal rabbit anti-DYRK1A antibody (Cat #: sc-28899, Santa cruz biotechnology, Santa cruz, CA) was used as a primary antibody at a dilution of 1:50. The sections were incubated overnight with primary antibody at 4 °C. Then the sections were rinsed with wash buffer followed by incubation with rabbit secondary antibody conjugated horseradish peroxidase (Merck-Banglore Genei, India). The staining was developed for 5 min using DAB chromogen (Dako, Glostrup, Denmark), followed by counterstaining with hematoxylin (Nice Chemicals, Kochi, India). The tissue sections were then examined under the microscope by pathologist and the immunohistochemical labeling was assessed to score the intensity of staining. The staining was scored on a scale of 0–2, where 0 represented absent staining, +1 represented faint staining and +2 represented intense staining. Cases that were +2 were considered as highly stained cases. A chi-square test was carried out to determine the significance of the immunohistochemistry results. Xenograft tissue sections were examined for the expression of Ki67 marker by immunofluorescence and Western blot analysis.

### Western blot analysis

Cells were cultured to 70% confluency and the proteins were harvested in RIPA lysis buffer (10 mM Tris pH 7.4, 150 mM NaCl, 5 mM EDTA, 1% Triton-X-100, 0.1% SDS containing protease and phosphatase inhibitor cocktails) and sonicated. Western blot analysis was carried out as described previously^45^,^48^. Polyclonal rabbit anti-DYRK1A antibody was obtained from Santa cruz (Santa cruz biotechnology). Anti- FOXO3A, anti-AKT, anti-phospho FOXO3A (Ser 253) and anti-phospho-AKT (Ser 473) antibodies were obtained from Cell Signaling Technology (Cell Signaling Technology, Beverly, MA). Beta-actin antibody was obtained from Sigma (St. Louis, MO).

### Annexin V/PI staining

Apoptosis was determined by staining cells with annexin Vfluorescein isothiocyanate (FITC) and PI labeling as per manufacturer’s instructions (Becton Dickinson, Franklin Lakes, NJ). Briefly, JHU-O28 and CAL 27 cells were treated with or without harmine. The prepared cells were washed twice with cold PBS, resuspended in 1 ml of binding buffer. 100 ul of the resuspended culture is stained with Annexin V and PI. The cells were analyzed on the BD FACSVerse^TM^ flow cytometer (Becton Dickinson, Franklin Lakes, NJ). Data analysis was done using “FlowJo” software.

## Additional Information

**How to cite this article**: Radhakrishnan, A. *et al*. A dual specificity kinase, DYRK1A, as a potential therapeutic target for head and neck squamous cell carcinoma. *Sci. Rep.* **6**, 36132; doi: 10.1038/srep36132 (2016).

**Publisher’s note:** Springer Nature remains neutral with regard to jurisdictional claims in published maps and institutional affiliations.

## Supplementary Material

Supplementary Information

## Figures and Tables

**Table 1:** A partial list of hyperphosphorylated proteins in at least four cell lines.

Gene Symbol	Phosphopeptide Sequence	Protein Description	PhosphoSite (Protein)	JHU-O28/OKF6/TERT1	JHU-O11/OKF6/TERT1	FaDu/OKF6/TERT1	CAL 27/OKF6/TERT1	JHU-O22/OKF6/TERT1	JHU-O29/OKF6/TERT1
*CDK1*	IGEGtyGVVYK	Cyclin-dependent kinase 1	T14; Y15	26.6	8.4	11.8	8.3	16.3	13.2
*CAV1*	YVDSEGHLyTVPIR	Caveolin-1	Y14	1.2	1.7	2.9	3.7	1.1	1.8
*LYN*	VIEDNEYtAR	Tyrosine-protein kinase Lyn	T377	1.8	2.6	9.7	6.6	4.4	6.9
*EGFR*	GSTAENAEyLR	Epidermal growth factor receptor	Y1197	1.0	1.6	5.7	19.9	3.7	7.1
*MPZL1*	SESVVyADIR	Myelin protein zero like protein 1	Y113	6.4	1.5	3.2	2.1	1.1	4.1
*EPHA2*	TYVDPHTyEDPNQAVLK	Ephrin type-A receptor 2	Y594	6.7	5.1	3.2	6.7	4.3	7.3
*PTPN11*	GHEyTNIK	Tyrosine-protein phosphatase non-receptor type 11	Y542	2.9	1.4	2.3	3.1	2.5	2.8
*DYRK1A*	IYQyIQSR	Dual specificity tyrosine-phosphorylation-regulated kinase 1A	Y321	1.8	2.7	3.8	2.1	2.9	3.8
*CTNND1*	SLDNNySTPNER	Catenin delta-1	Y797	0.2	1.2	4.3	3.6	1.7	7.0
*ANXA2*	SYSPyDMLESIR	Annexin A2	Y238	0.6	1.6	7.7	0.9	1.8	3.2

**Table 2:** Summary of the immunohistochemical validation of DYRK1A in HNSCC and normal tissues.

Staining Intensity	Tumor cases	Normal cases
Strong	25	1
Moderate	14	5
Negative	1	2
p-value of significant difference between tumor and normal groups (Chi-square test)	0.0076	
