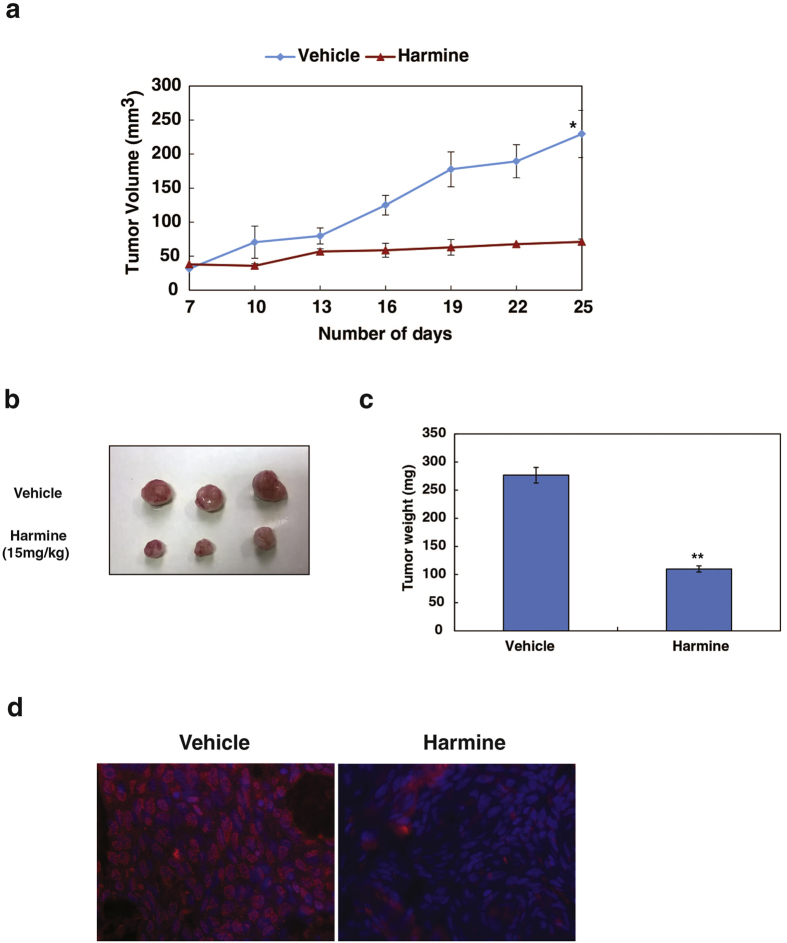# Corrigendum: A dual specificity kinase, DYRK1A, as a potential therapeutic target for head and neck squamous cell carcinoma

**DOI:** 10.1038/srep46864

**Published:** 2017-06-26

**Authors:** Aneesha Radhakrishnan, Vishalakshi Nanjappa, Remya Raja, Gajanan Sathe, Vinuth N. Puttamallesh, Ankit P. Jain, Sneha M. Pinto, Sai A. Balaji, Sandip Chavan, Nandini A. Sahasrabuddhe, Premendu P. Mathur, Mahesh M. Kumar, T. S. Keshava Prasad, Vani Santosh, Geethanjali Sukumar, Joseph A. Califano, Annapoorni Rangarajan, David Sidransky, Akhilesh Pandey, Harsha Gowda, Aditi Chatterjee

Scientific Reports 6: Article number: 36132; 10.1038/srep36132 published online: 10 31 2016; updated: 06 26 2017.

This Article contains errors. The Acknowledgements section is incomplete.

“We thank the Department of Biotechnology (DBT), Government of India for research support to the Institute of Bioinformatics (IOB), Bangalore. We thank the “Infosys Foundation” for research support to IOB. AC was supported by Department of Science and Technology (DST) grants (SERC/LS-439/2011 and SR/SO/HS/0208/2013). IOB is supported by DBT Program Support on Neuroproteomics and infrastructure for proteomic data analysis (BT/01/COE/08/05). AR, GS and SC are recipients of Senior Research Fellowship from Council of Scientific and Industrial Research (CSIR), Government of India. RR is a recipient of research associateship from DBT. We thank Dr. S. K. Shankar and Dr. Anita Mahadevan of National Institute of Mental Health and Neurological Sciences (NIMHANS), for providing access to the microscopy imaging facility. We thank Dr. V. Ravi for providing access to advanced FACS facility in NIMHANS. We thank Ms. Neha Deshpande for technical inputs with fluorescence imaging”.

should read:

“We thank the Department of Biotechnology (DBT), Government of India for research support to the Institute of Bioinformatics (IOB), Bangalore. We thank the “Infosys Foundation” for research support to IOB. AC was supported by Department of Science and Technology (DST) grants (SERC/LS-439/2011 and SR/SO/HS/0208/2013) and FAMRI-funded 072017_YCSA. IOB is supported by DBT Program Support on Neuroproteomics and infrastructure for proteomic data analysis (BT/01/COE/08/05). AR, GS and SC are recipients of Senior Research Fellowship from Council of Scientific and Industrial Research (CSIR), Government of India. RR is a recipient of research associateship from DBT. We thank Dr. S. K. Shankar and Dr. Anita Mahadevan of National Institute of Mental Health and Neurological Sciences (NIMHANS), for providing access to the microscopy imaging facility. We thank Dr. V. Ravi for providing access to advanced FACS facility in NIMHANS. We thank Ms. Neha Deshpande for technical inputs with fluorescence imaging”.

Additionally in Figure 4b, the label ‘Harmine (15 mg/kg)’ is incorrectly given as ‘Harmine (30 mg/kg)’. The correct Figure 4 appears below as Figure 1.

## Figures and Tables

**Figure 1:**