# Supplementary material for: A dual specificity kinase, DYRK1A, as a potential therapeutic target for head and neck squamous cell carcinoma
Source: Sci Rep. 2016 Oct 31;6:36132. doi: 10.1038/srep36132 (PMC5086852; doi:10.1038/srep36132)

A dual specificity kinase, DYRK1A, as a potential therapeutic target for head and neck squamous cell carcinoma

Aneesha Radhakrishnan[1,2], Vishalakshi Nanjappa[1,3], Remya Raja[1], Gajanan Sathe[1,4], Vinuth N. Puttamallesh[1,3], Ankit P. Jain[1,5], Sneha M. Pinto[1], Sai A. Balaji[6], Sandip Chavan[1,4], Nandini A. Sahasrabuddhe[1], Premendu P. Mathur[2,5], Mahesh M. Kumar[7], T. S. Keshava Prasad[1,3,8], Vani Santosh[9], Geethanjali Sukumar[1], Joseph A. Califano[10,11], Annapoorni Rangarajan[6], David Sidransky[11], Akhilesh Pandey[12,13,14,15], Harsha Gowda[1,8]*, Aditi Chatterjee[1,8]*

[1]Institute of Bioinformatics, International Technology Park, Bangalore, 560 066, India

[2]Department of Biochemistry and Molecular Biology, Pondicherry University, Puducherry 605014, India

[3]Amrita School of Biotechnology, Amrita University, Kollam 690 525, India

[4]Manipal University, Madhav Nagar, Manipal 576104, India

[5]School of Biotechnology, KIIT University, Bhubaneswar 751024, India

[6]Department of Molecular Reproduction, Development and Genetics, Indian Institute of Science, Bangalore, 560012, India

[7]Department of Neuro-Virology, National Institute of Mental Health and Neurosciences, Bangalore 560029, India

[8]YU-IOB Center for Systems Biology and Molecular Medicine, Yenepoya University, Mangalore 575018, India

[9]Department of Pathology, National Institute of Mental Health and Neurosciences, Bangalore 560029, India

[10]Milton J. Dance Head and Neck Center, Greater Baltimore Medical Center, Baltimore, MD 21204, USA

[11]Department of Otolaryngology-Head and Neck Surgery, Johns Hopkins University School of Medicine, Baltimore, MD 21231, USA

[12]McKusick-Nathans Institute of Genetic Medicine, [13]Departments of Biological Chemistry, [14]Oncology and [15]Pathology, Johns Hopkins University School of Medicine, Baltimore, MD 21205, USA

[*]To whom correspondence should be addressed:

Aditi Chatterjee, Ph.D.

Institute of Bioinformatics,

International Tech Park,

Bangalore-560 066, India

E-mail: aditi@ibioinformatics.org

Harsha Gowda, Ph.D.

Institute of Bioinformatics,

International Tech Park,

Bangalore-560 066, India

E-mail: harsha@ibioinformatics.org

Supplementary Figure legends

Supplementary figure 1

Experimental workflow employed for mass spectrometry analysis.
Equal amount of protein from each cell line were extracted, digested and labeled with TMT reagents. The labeled samples were pooled and purified using Sep-Pak C_{18} columns. The purified sample was enriched for tyrosine using phosphotyrosine antibody. The phosphotyrosine enriched samples were analyzed in Orbitrap velos mass spectrometer. The MS/MS data was searched against Refseq 65 protein database using SEQUEST and Mascot search algorithms.

Supplementary figure 2

Effect of DYRK1A inhibition using harmine on HNSCC cell proliferation (*$p<0.05$)

Experimental workflow employed for mass spectrometry analysis

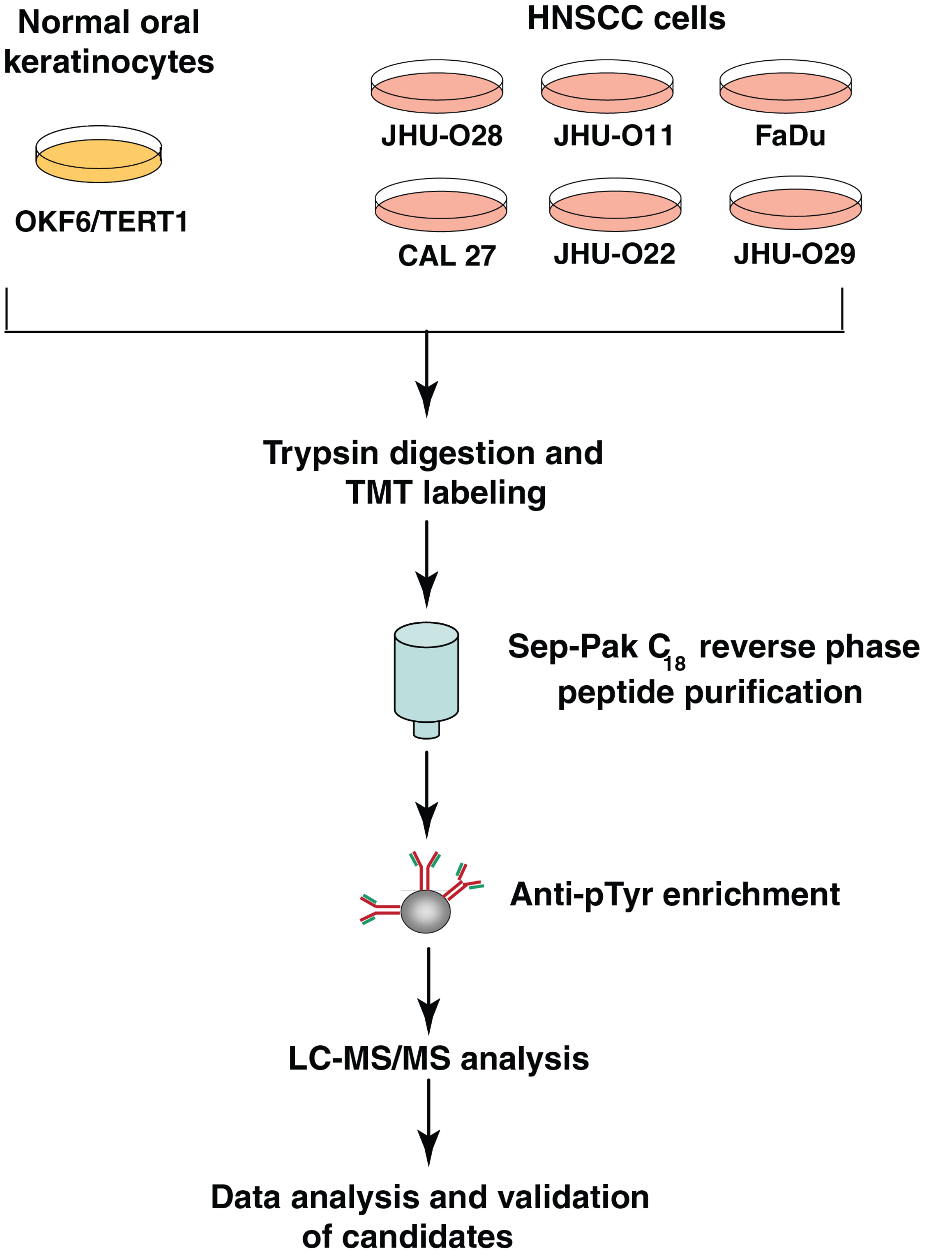

Effect of DYRK1A inhibition using harmine on HNSCC cell proliferation

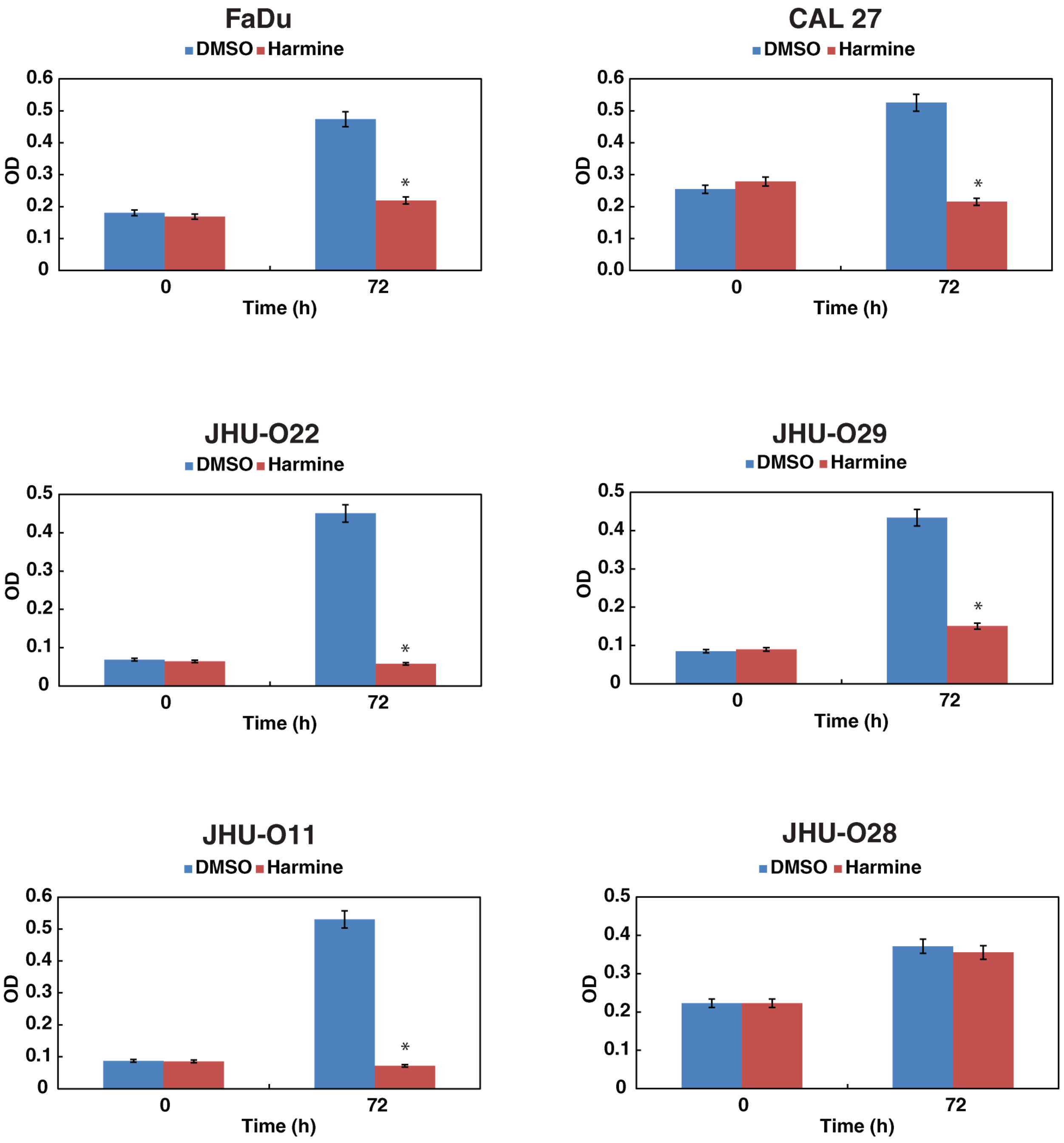

Supplement: Supplementary Information [file srep36132-s1.pdf]
